# Transcription factor TCF4: structure, function, and associated diseases

**DOI:** 10.18699/vjgb-24-85

**Published:** 2024-11

**Authors:** R.R. Savchenko, N.A. Skryabin

**Affiliations:** Research Institute of Medical Genetics, Tomsk National Research Medical Center of the Russian Academy of Sciences, Tomsk, Russia; Research Institute of Medical Genetics, Tomsk National Research Medical Center of the Russian Academy of Sciences, Tomsk, Russia

**Keywords:** TCF4, Pitt–Hopkins syndrome, bHLH, mental disorders, autism spectrum disorders, Pitt–Hopkins syndrome therapy, TCF4, синдром Питта–Хопкинса, bHLH, психические расстройства, расстройства аутистического спектра, терапия синдрома Питта–Хопкинса

## Abstract

Our understanding of human genes – particularly their structure, functions, and regulatory mechanisms – is still limited. The biological role of approximately 20 % of human proteins has not been established yet, and the molecular functions of the known part of the proteome remain poorly understood. This hinders progress in basic and applied biological and medical sciences, especially in treating hereditary diseases, which are caused by mutations and polymorphic variants in individual genes. Therefore, it is crucial to comprehend the mechanisms of protein functioning to address this problem. This further emphasizes the importance of investigating gene functions and molecular pathogenetic pathways associated with single-gene inherited diseases. This review focuses on the TCF4 gene that encodes a transcription factor crucial for nervous system development and functioning. Pathogenic variants in this gene have been linked to a rare genetic disorder, Pitt–Hopkins syndrome, and TCF4 polymorphic variants are associated with several socially significant diseases, including various psychiatric disorders. The pathogenetic mechanisms of these conditions remain unexplored, and the knowledge about TCF4 upregulation and its target genes is limited. TCF4 can be expressed in various isoforms due to the complex structure and regulation of its gene, which complicates the investigation of the protein’s functions. Here, we consider the structure and functions of the TCF4 transcription factor. We discuss its potential target genes and the possible loss-of-function pathogenetic mechanisms identified in animal and cellular models of Pitt–Hopkins syndrome. The review also examines the advantages and limitations of potential therapies for Pitt–Hopkins syndrome that are based on TCF4 dosage compensation or altering the activity of TCF4 target genes.

## Abstract (ru)

На сегодняшний день имеются ограниченные знания об основных характеристиках генов человека, их структуре, функции и механизмах регуляции экспрессии. Биологическая роль около 20 % белковых продуктов генов до сих пор не установлена, а молекулярные функции известной части протеома остаются недостаточно изученными. Данное обстоятельство ограничивает прогресс как фундаментальных, так и прикладных биологических и медицинских наук, в особенности в случае терапии наследственных болезней, патогенез которых обусловлен наличием вариантов в нуклеотидной последовательности отдельных генов. В связи с этим возрастает необходимость проведения исследований, направленных на изучение функций генов, а также молекулярных патогенетических путей, связанных с развитием моногенных заболеваний. Наша статья посвящена гену TCF4, кодирующему широко экспрессируемый фактор транскрипции, важный для развития и функционирования нервной системы. К настоящему времени установлено, что патогенные варианты в этом гене приводят к развитию редкого генетического заболевания, известного как синдром Питта–Хопкинса, а полиморфные варианты в TCF4 ассоциированы с рядом социально значимых заболеваний, представленных различными психическими расстройствами. Молекулярные механизмы патогенеза подобных состояний по-прежнему остаются неизученными, а знания о вышестоящей регуляции TCF4 и его нижестоящих генах-мишенях ограничены. Сложность структурной организации и особенности регуляции экспрессии гена обеспечивают многообразие изоформ TCF4, что затрудняет понимание молекулярных функций белка. В обзоре рассмотрены известные данные о структуре и функциях фактора транскрипции TCF4. Обсуждаются потенциальные гены-мишени и возможные патогенетические механизмы, обусловленные потерей функции этого белка, выявленные в исследованиях на животных и клеточных моделях синдрома Питта–Хопкинса. Рассмотрены преимущества и ограничения потенциальных стратегий терапии указанного синдрома, основанные на компенсации дозы TCF4 или воздействии на молекулярные мишени изучаемого транскрипционного фактора.

## Introduction

One of the most important problems in medical genetics today is the limited understanding of the role of proteins involved in the molecular pathways underlying the development of several inherited disorders. This problem is especially urgent regarding transcription factors, since these proteins regulate the expression of many genes, have pleiotropic effects, and are critical for various biological processes. One such transcription factor is encoded by the TCF4 gene. Pathogenic variants in this gene are responsible for Pitt–Hopkins syndrome development (Amiel et al., 2007; Brockschmidt et al., 2007; Zweier et al., 2007), and its polymorphic variants have been associated with various psychiatric disorders including schizophrenia, bipolar disorder, major depressive disorder, and post-traumatic stress disorder (Stefansson et al., 2009; Smoller et al., 2013; Wray et al., 2018).

According to the literature, TCF4 is critical for brain development and function, as it participates in nerve cell differentiation and migration, regulation of neuronal excitability, neuronal plasticity, etc. (Imayoshi, Kageyama, 2014; Kennedy et al., 2016; Li H. et al., 2019; Mesman et al., 2020; Phan et al., 2020). Despite the large amount of data pointing to the important role of TCF4, the molecular mechanisms of its pathogenic variants leading to impaired development and function of cells in the nervous system remain largely unexplored. This review aims to summarize the literature data on TCF4 structure and function, its known molecular targets, diseases associated with variants in TCF4, and potential approaches to their therapy.

## TCF4 structure, expression pattern and known functions

The TCF4 gene, also known as ITF2 or PTHS, is located in the 18q21.2 region on chromosome 18 and contains 41 exons. Twenty of these exons are alternative 5′-exons (non-proteincoding exons that are included or excluded from mature mRNA to regulate the expression of longer or shorter protein isoforms), 20 exons are internal protein-coding, and one is a 3′-non-coding exon (Sepp et al., 2011). TCF4 encodes a transcription factor containing a basic helix-loop-helix structural motif (bHLH). The proteins of this group contain a DNAbinding domain and can regulate gene expression, forming homo- and heterodimers. The bHLH-containing proteins are categorized into six groups depending on the types of dimers they form, expression pattern, and DNA binding specificity. The transcription factor TCF4 belongs to the E-proteins group (or class I bHLH proteins) that recognize E-box sequences (CANNTG) located in the promoter and enhancer regions of target genes (Schoof et al., 2020). The bHLH domain is a highly conserved motif consisting of a basic amino acid region followed by two amphipathic α-helixes connected by a loop. The basic amino acid region binds to the E-box sequence directly, while the α-helices provide dimerization.

The alternative transcription initiation sites located upstream of non-coding exons 1, 3, 4, 5, 7, 8, and 10 define at least 18 TCF4 isoforms. The isoforms contain relatively conserved C-terminal domains of the basic helix-loop-helix structural motif but differ in N-terminal regions responsible for transcription regulation. However, it should be noted that the diversity of TCF4 transcripts is even higher due to alternative splicing of internal coding exons (Teixeira et al., 2021).

**Fig. 1.**
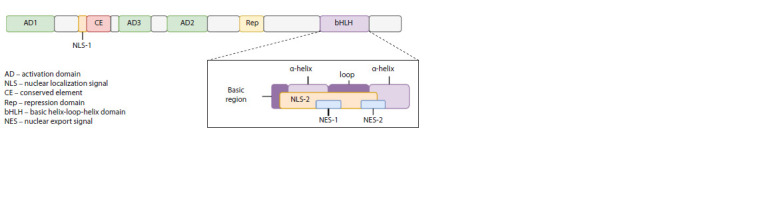
Schematic representation of the full-length TCF4 protein structure The figure is modified from J.R. Teixeira and colleagues (Teixeira et al., 2021).

Full-length TCF4 transcripts include the following structural elements: the bHLH domain, activation domains (AD1, AD2 and AD3), CE and Rep intramolecular regulatory domains, NLS-1 and NLS-2 nuclear localization signals, NES-1 and NES-2 nuclear export signals, and the RSRS sequence of four amino-acid residues (Fig. 1).

In addition to the bHLH domain, the AD1, AD2, and AD3 activation domains can cooperatively or independently regulate gene expression in a cell type-dependent manner. It has been shown that the AD1 domain can bind transcription coactivators and corepressors. The AD2 domain can bind coactivators of transcription, but no data are currently available on its interactions with corepressors. Transcription coactivators and corepressors compete for binding to the AD1 domain, enabling TCF4 to both activate and repress gene expression (Teixeira et al., 2021).

The AD3 domain interacts directly with the TAF4 subunit of the general transcription factor II D, resulting in enhanced RNA polymerase II preinitiation on target genes, but exactly how AD3 is involved in the regulation of gene expression by TCF4 is currently unclear (Teixeira et al., 2021).

The TCF4 transcriptional activity is also regulated by the previously mentioned CE and Rep domains. The conserved element (CE) located between the activation domains of AD1 and AD3 can inhibit AD1 activity. The repression domain (Rep), located between AD2 and bHLH, is able to repress the activity of AD1 and AD2. Both of these domains can likely prevent the recruitment of transcriptional cofactors, and as a result, suppress AD1-mediated activation or repression of transcription (Teixeira et al., 2021).

Finally, the presence of the RSRS four amino-acid residue motif (Arg-Ser-Arg-Ser) located between the Rep and bHLH domains may also result in reduced transcriptional activity

The complex structural organization of TCF4, along with the peculiar regulation of its expression, results in a variety of TCF4 isoforms containing different structural domains. Since all TCF4 transcripts include exons 10–20, all isoforms of the encoded protein contain the AD2 and AD3 activation domains, as well as the bHLH, Rep, NLS-2, NES-1, and NES- 2 domains. Only the four longer protein isoforms contain the full AD1 activation domain, while the other isoforms contain either only a part of it or none at all (Sepp et al., 2011). In addition, the literature describes “Δ-isoforms”, which are characterized by the absence of NLS-1 and CE domains. Finally, the presence of alternative splicing sites in exon 18 leads to the inclusion or exclusion of the segment encoding the RSRS sequence present in positive (+) isoforms and absent in negative (–) isoforms of the protein (Sepp et al., 2011). How various isoforms differ from each other in terms of transcription regulation remains an open question.

Similar to the majority of genes encoding E-proteins, TCF4 is expressed in almost all tissues of the organism, being the most abundant in the brain (The Human Protein Atlas, https:// www.proteinatlas.org). Some TCF4 transcripts are tissuespecific, while others have a broad spatial expression pattern. Moreover, the quantitative ratios of the same transcripts can vary in different tissues. The expression analysis using RTPCR showed that most TCF4 transcripts were expressed in the brain, except for the five found in the testes (Sepp et al., 2011). TCF4 expression is also upregulated during ontogenesis, with the highest activity during prenatal development (Sepp et al., 2011). It has been shown that TCF4 expression in the brain increases toward the end of the prenatal period and then decreases to baseline in newborns, persisting throughout life (Li M. et al., 2018).

In humans, TCF4 is expressed in the forebrain and brain ventricular system during fetal development and persists in the forebrain and cerebellum in adults. Besides, TCF4 is found in oligodendrocytes of the spinal cord (Chen H.Y. et al., 2021).

The variety of TCF4 isoforms complicates the understanding of its molecular functions. The functions of specific TCF4 isoforms depend on which 5′-exon and internal exons are included in the translated transcript. Depending on the isoform structure, both subcellular localization and transcription are differentially regulated. For example, isoforms containing NLS are localized in the nucleus, whereas isoforms lacking NLS require a heterodimerization partner to access the nucleus (Chen H.Y. et al., 2021).

As a transcription factor, TCF4 has been associated with the regulation of hematopoiesis, myogenesis, neurogenesis, melаnogenesis, osteogenesis, and the differentiation of endothelial, mammary, and Sertoli cells (Teixeira et al., 2021). Additionally, TCF4 appears critical for normal nervous system development and function. This protein forms heterodimers with the transcription factors ATOH1, ASCL1, NEUROD1, and NEUROD2, which play an important role in nervous system development (Wittmann, Häberle, 2018). TCF4 is known to be important for brain development and functioning: it participates in such processes as the differentiation of neuronal progenitor cells into neurons, oligodendrocyte and astrocyte (Imayoshi, Kageyama, 2014), maturation, neuronal migration and function, oligodendrocyte myelination, synaptic plasticity, etc. (Kennedy et al., 2016; Li H. et al., 2019; Mesman et al., 2020; Phan et al., 2020).

## TCF4-associated diseases

To date, a number of studies have indicated a possible role for TCF4 in the pathogenesis of various socially important diseases. Genome-wide association studies show that polymorphic variants in TCF4, predominantly localized in non-coding regions of the gene, are associated with various psychiatric disorders, including schizophrenia (Stefansson et al., 2009; Ripke et al., 2011; Steinberg et al., 2011; Smoller et al., 2013; Bocharova et al., 2017), bipolar disorder and autism spectrum disorders (Smoller et al., 2013), major depressive disorder (Wray et al., 2018), and post-traumatic stress disorder (Gelernter et al., 2019). In addition, variants in TCF4 are associated with Fuchs’ corneal endothelial dystrophy (Afshari et al., 2017; Fautsch et al., 2021) and sclerosing cholangitis (Ellinghaus et al., 2013). However, it is currently unknown whether these polymorphic variants are responsible for the development of these diseases. The exception is Fuchs’ endothelial corneal dystrophy: most patients with this diagnosis carry an expansion of the trinucleotide repeat (CTG)n in intron 3 of the TCF4 gene, leading to splicing errors (Du et al., 2015; Papanyan et al., 2019) (see the Table).

**Table 1.**
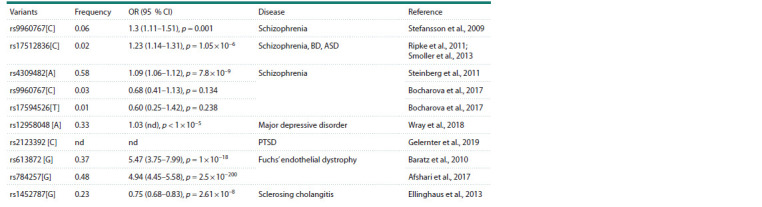
Diseases associated with polymorphic variants in TCF4 Note. OR – odds ratio; CI – confidence interval; BD – bipolar disorder; ASD – autism spectrum disorders; PTSD – post-traumatic stress disorder; nd – no data.

In 2007, several independent studies showed that heterozygous carriage of pathogenic variants in the TCF4 gene leads to the development of a rare inherited disease, Pitt–Hopkins syndrome (PTHS) (Amiel et al., 2007; Brockschmidt et al., 2007; Zweier et al., 2007). Despite phenotypic differences, most patients with this syndrome are characterized by a specific set of dysmorphic facial features combined with intellectual disability, sensorimotor impairment, speech delay, and generalized muscular hypotonia. About 78 % of patients frequently perform stereotypical and intense repetitive movements, which allows PTHS to be classified as an autism spectrum disorder. Approximately half of patients with PTHS have abnormal breathing patterns and about one-third develop epileptic seizures. In addition, magnetic resonance imaging has identified several brain anomalies in patients with PTHS, including agenesis of the corpus callosum, large ventricles, and an abnormal shape of the posterior cranial fossa (Teixeira et al., 2021).

The spectrum of TCF4 mutations identified in patients with PTHS includes missense (~15 % of cases), nonsense (~15 %), splicing site mutations (~10 %), small insertions or deletions resulting in frame shifts (~30 %), translocations and large deletions encompassing TCF4 partially or fully (~30 %) (Teixeira et al., 2021). Some estimates put the worldwide prevalence of PTHS caused by chromosomal deletions at 1/34,000–1/41,000 (Rosenfeld et al., 2009).

Depending on the localization and mutation type, TCF4 isoforms are affected differently. The majority of missense mutations affect exon 19 encoding the bHLH domain. Certain missense mutations affect exons 15 and 18 encoding the AD2 activation domain and the Rep regulatory domain, respectively. Since all TCF4 transcripts contain these exons, the pathogenic variants described lead to the disruption of all protein isoforms. Most nonsense, frame-shift, and splice-site mutations also result in damage to all isoforms of the protein. However, if these mutations occur in exons 8 and 9 or are localized upstream of exons 10a-c, the Δ-isoforms and shorter TCF4 isoforms are unchanged. Several translocations and deletions span only initial exons (1 through 4) or inner exons (5 through 9), retaining intermediate and shorter isoforms, respectively (Sepp et al., 2012).

The effects of the structural diversity and cell-specific TCF4 expression pattern on physiologic processes remain poorly understood. However, it is hypothesized that different types of TCF4 mutations in individuals with PTHS may impair the functions of the encoded protein by diverse mechanisms and to a varying extent, thus leading to the phenotypic variability observed among patients (Bedeschi et al., 2017). For example, missense mutations in the bHLH motif or insertions elongating the reading frame can damage DNA-binding or transactivation functions in a manner dependent on dimer context (Sepp et al., 2012). Pathogenic variants encompassing the bHLH domain responsible for dimerization destabilize the protein, whereas missense mutations outside of the bHLH domain cause no major functional deficiencies (Chen H.Y. et al., 2021).

The majority of the pathogenic variants in TCF4 found in patients with PTHS lead to haploinsufficiency because they restrict the expression of certain or all transcripts to a single copy of the allele. In addition, certain missense mutations cause the attenuation or loss of TCF4 function as a transcription regulator without affecting its ability to dimerize in vitro, which seems to indicate a dominant-negative effect (Forrest et al., 2013). Whether the observed effect occurs in vivo is currently unclear. Presumably, it would be weak due to dimer instability with mutant TCF4 (Teixeira et al., 2021). Thus, it is evident that PTHS results from the dysregulation of TCF4- mediated gene expression. How such disturbances can trigger a pathophysiologic process remains unclear. J.R. Teixeira et al. (2021) suggest this process may be related to the general functions of E-proteins during the regulation of the cell cycle and to the specific role of TCF4 in cell differentiation

## Molecular pathways and potential target genes regulated by TCF4

**Fig. 2.**
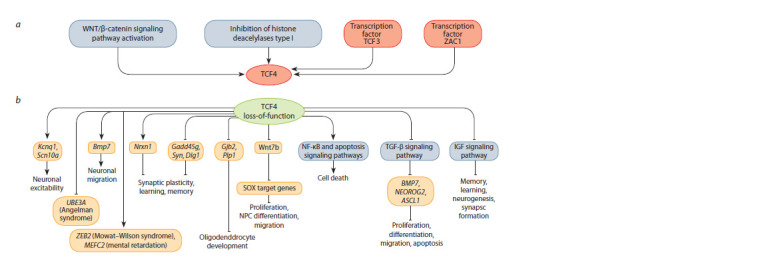
Upregulation of TCF4 and its potential molecular targets. a – upregulation of TCF4; b – molecular pathways and potential target genes regulated by TCF4. NPC – neural progenitor cells. Pointed-end arrows indicate activation and blunt-end arrows indicate inhibition.

To date, there has been progress in identifying upstream regulators and target genes of the TCF4 transcription factor. K.M. Henning et al. showed that the pharmacological activation of the WNT/β-catenin signaling pathway in induced pluripotent stem cells (iPSCs), derived from neural progenitor cells and neurons from patients with PTHS, leads to increased TCF4 expression (Hennig et al., 2017). Chromatin modification mediated by the inhibition of class I histone deacetylases has a similar effect (Kennedy et al., 2016; Hennig et al., 2017). TCF3, a member of the E-protein subgroup, and the ZAC1 transcription factor also upregulate Tcf4 expression (Schmidt- Edelkraut et al., 2014; Li H. et al., 2019). The authors suggest that Tcf4 regulation by TCF3 and other unidentified transcription factors is crucial for normal cortical development (Li H. et al., 2019) (Fig. 2).

Using ChIP-Seq technology, several studies have identified direct TCF4 targets, including Bmp7 (Chen T. et al., 2016), Nrxn1 (D’Rozario et al., 2016), Gadd45g (Sepp et al., 2017), Gjb2, and Plp1 (Wedel et al., 2020). Cellular and animal models have helped identify the following molecular targets of TCF4: Scn10a (Nav1.8) and Kcnq1 (Kv7.1) (Rannals et al., 2016; Martinowich et al., 2022), Wnt7b (Wang et al., 2020), Gadd45g (Tamberg et al., 2020), Syn and Dlg1 (Tamberg et al., 2020). Collectively, these studies indicate that TCF4 regulates genes involved in brain development, nerve cell differentiation, neuronal excitability, synapse function, and survival (Fig. 2).

However, according to the literature, TCF4 has more than ten thousand binding sites in the genome, potentially connected to more than five thousand genes (Forrest et al., 2018; Xia et al., 2018). In light of these data, it is clear that the vast majority of molecular targets of this transcription factor remain unidentified. Therefore, identifying molecular pathways and target genes regulated by TCF4 is essential for fundamental research of the processes controlled by transcription factors. Moreover, understanding of the mechanisms of the gene network function and identification of the key molecular targets of TCF4 may significantly influence the development of therapeutic strategies for TCF4-associated diseases.

To date, several studies using animal models have assessed changes in the transcriptional profile caused by TCF4 mutations. The genes encoding potassium and sodium ion channels, Kcnq1 and Scn10a, are identified as downstream targets of TCF4 in rodent models (Rannals et al., 2016; Martinowich et al., 2022). Both studies demonstrate overexpression of these genes coupled with the loss of TCF4 function, which allows to consider this transcription factor as a regulator of neuronal excitability. Other studies have reported downregulation of the Arc gene, which is important for synaptic plasticity, information processing, and memory (Kennedy et al., 2016), and the Wnt7b gene, which is considered a key TCF4 target in the regulation of neuronal progenitor cell migration during dentate gyrus development (Wang et al., 2020) (Fig. 2).

Several studies demonstrate that mice with Tcf4 mutations are characterized by an increased expression of genes associated with neuronal progenitor cell proliferation and a suppressed expression of genes involved in neuronal differentiation and migration (Li H. et al., 2019), neurogenesis, and neuronal maturation (Mesman et al., 2020). B.D.N. Phan et al. (2020) also reported an abnormal gene expression pattern in oligodendrocytes, in particular the genes involved in myelination, which is critical for the normal function of these cells (Fig. 2).

Thus, animal research suggests that TCF4 is crucial for brain development and function and identifies potential targets of this transcription factor. However, animal model systems have significant limitations when it comes to extrapolating their results to humans. For instance, TCF4 haploinsufficiency is known to result in clinical manifestations of PTHS in patients, whereas heterozygous mice carrying Tcf4 mutations (wt/Tcf4–) tend to exhibit milder phenotypes (Thaxton et al., 2018; Li H. et al., 2019; Mesman et al., 2020; Wang et al., 2020). These differences appear to be due to significant differences between the structure and development of rodent and human brains, which should be taken into account. This fact highlights the need for research to be conducted on human nerve cells.

In the study by F. Papes et al. (2022), fibroblasts derived from patients with PTHS were reprogrammed into iPSCs with subsequent differentiation into neural progenitor cells, neurons, and brain organoids. The authors showed that neuronal progenitor cells with TCF4 mutations were characterized by reduced proliferation and impaired neuron differentiation, while brain organoids were characterized by abnormal size and cellular composition (Papes et al., 2022). Based on the RNA sequencing results, the authors suggest that the loss of TCF4 function leads to disruptions of the Wnt signaling pathway, and, as a result, a decreased expression of SOX target genes, ultimately leading to the reduced proliferation of progenitor cells (Papes et al., 2022). The rescue of TCF4 expression or pharmacological correction of the Wnt signaling pathway resulted in a partial recovery of aberrant phenotypes. These data indicate possible therapeutic strategies for TCF4-associated genetic disorders.

Several studies using SH-SY5Y to model TCF4 dysfunction are found in the literature and provide valuable insight into the molecular mechanisms regulated by this transcription factor. The microarray analysis of the transcriptional profile performed in the SH-SY5Y cells with TCF4 knockdown revealed differentially expressed genes (DEGs) involved in the TGFβ signaling pathway, epithelial-mesenchymal transition, neuronal differentiation, and apoptosis (Forrest et al., 2013). The genes encoding EMT, SNAI2, and DEC1 transcription factors, as well as NEUROG2 and ASCL1 proneural genes, and genes associated with intellectual disability, such as UBE3A (Angelman syndrome), ZEB2 (Mowat–Wilson syndrome), were characterized by the most pronounced differential expression. The findings suggest that TCF4 regulates several molecular pathways associated with nerve cell differentiation and survival, as well as genes clinically significant to the pathogenesis of intellectual disability.

In another study, H. Xia et al. applied ChIP-seq techno- logy in SH-SY5Y cells to analyze DNA-protein interactions and detect TCF4-binding sites (Xia et al., 2018). This approach has identified more than 10,000 binding sites that can be attributed to more than 5,500 genes. The gene set enrichment analysis (GSEA) of potential target genes revealed the pathways associated with neuronal develop- ment and identified genes that overlap with those underexpressed postmortem in the brains of patients with schizophrenia. These data further support the importance of TCF4 for brain development and function and indicate the existence of pathogenetic molecular pathways common to PTHS and schizophrenia (Xia et al., 2018).

Thus, studies conducted in animal models of PTHS have identified variability in the phenotypes that provide important biological information about this disorder. The phenotypes described above are observed throughout life, ranging from abnormalities in cortical development and nerve cell differentiation and maturation to impairments in neuronal excitability, synaptic plasticity, and behavior in adult animals. Although the analyses of transcriptional profiles using microarray and RNA sequencing do not point directly to TCF4 target genes, they emphasize the important role of this transcription factor in neurogenesis and demonstrate a large-scale gene network potentially regulated by TCF4. Understanding of the molecular pathways and identification of TCF4 target genes are crucial for comprehending the pathogenesis of TCF4-associated disorders and identifying potential therapeutic targets.

## Potential therapeutic strategies for Pitt–Hopkins syndrome

Pitt–Hopkins syndrome patients require lifelong medical care, but current therapeutic approaches focus on symptomatic treatment. Although there is currently no effective treatment for PTHS, research is ongoing to understand the molecular mechanisms behind the disease and to identify potential therapeutic targets. Several potential therapeutic approaches have been tested in preclinical mouse models of PTHS. The first approach corrects gene transcriptional activity using histone deacetylase inhibitors, which have been associated with improved memory and learning ability. The administration of histone deacetylase inhibitor SAHA improved cognitive function and memory in mice with heterozygous Tcf4 mutations (a deletion of exons encoding the bHLH domain) (Kennedy et al., 2016). Other studies have selected the sodium potentialdependent NaV1.8 channel encoded by the SCN10A gene as a therapeutic target. TCF4 loss-of-function leads to ectopic overexpression of Scn10a, and the pharmacological inhibition of NaV1.8 in murine models of PTHS is effective for the restoration of several physiological functions and behavior (Ekins et al., 2020; Cleary et al., 2021; Martinowich et al., 2022). Specifically, S. Ekins and colleagues used Nicardepine, a drug approved by the Food and Drug Administration (FDA) and used in cardiology, as a NaV1.8 inhibitor (Ekins et al., 2020). Other selective NaV1.8 inhibitors have also been proven safe for humans in clinical trials (Hijma et al., 2021, 2022). Given these facts, testing NaV1.8 antagonists for PTHS therapy has significant potential.

The strategies discussed employ either upstream regulators of TCF4 activity or downstream target genes as therapeutic targets. Despite the success of these approaches in animal models, they have some limitations. The effects on upstream regulators of TCF4 are likely to lack specificity and entail undesirable adverse reactions arising from off-target transcriptional effects. The limitations of the second approach stem from the fact that TCF4 regulates the expression of hundreds or thousands of other genes (Forrest et al., 2013; Hill et al., 2017; Xia et al., 2018; Torshizi et al., 2019), which greatly complicates the identification of the key transcription modifier genes and the correction of their expression levels.

Since TCF4 loss-of-function underlies the disease, it can be hypothesized that rescuing gene expression using antisense oligonucleotides or gene therapy may prove to be the most effective treatment approach. However, given that TCF4 expression in humans peaks in the prenatal period and then decreases to the baseline level maintained throughout life (Rannals et al., 2016; Phan et al., 2020), the question arises about the possibility of restoring physiological and behavioral functions of patients with PTHS by normalizing TCF4 expression in the postnatal period. Moreover, it remains unclear to what extent TCF4 expression should be upregulated. The regulation of TCF4 dosage is extremely important because the disease can develop from either too low or too high expression levels. Pathogenic variants in TCF4 leading to haploinsufficiency may cause neurodevelopmental disorders, whereas polymorphic variants localized in non-coding regions of the gene lead to its overexpression and appear to be associated with schizophrenia.

A recent study by H. Kim et al. (2022) using a mouse model of PTHS showed that the development of the phenotypes characteristic of this syndrome can be prevented or partially corrected by normalizing Tcf4 expression, with the success of therapeutic intervention depending on the timing of exposure and cell type specificity. Pancellular rescue of Tcf4 expression in the prenatal period completely prevented the development of PTHS phenotypes. Selective restoration of gene expression in excitatory or inhibitory neurons during embryogenesis resulted in the rescue of a number of behavioral functions. Finally, postnatal restoration of Tcf4 expression using adenoassociated viral vectors in neurons reduced anxiety-like behavior, stimulated activity, and improved innate behaviors and memory. In addition, this approach led to a partial recovery of EEG parameters and correction of the expression levels of several Tcf4 target genes (Kim et al., 2022).

Gene therapy based on viral vectors holds great promise for the treatment of diseases previously considered incurable. According to the Gene Therapy Clinical Trials Worldwide database as of March 2023, the vectors based on adenoviruses, retroviruses, lentiviruses, and adeno-associated viruses were the most frequently used in clinical trials (https://a873679. fmphost.com/fmi/webd/GTCT; accessed 29.06.2024). Viralbased vectors have their advantages as well as undesirable effects. The latter include immune response, cytotoxicity, risks of genomic integration, and risks associated with the emergence of de novo replicative-competent viruses (Ertl, 2022; Leikas et al., 2023; Lundstrom, 2023).

Thus, gene therapy approaches to rescuing TCF4 expression developed in animal models may be effective for patients with PTHS. In this regard, further studies are needed to determine whether restoration of Tcf4 expression at different periods of ontogenesis can help correct behavioral and physiological dysfunction. The results of such studies may help us evaluate the effectiveness of therapy for different age groups of PTHS patients. In addition, potential therapy strategies using TCF4 expression level correction will have to ensure appropriate biodistribution of the encoded protein, as studies show that restoration of gene activity only in certain cells and brain structures can lead to the normalization of some behavioral and physiological functions in laboratory animals (Kim et al., 2022). One of the main advantages of gene therapy for PTHS is that it does not require an understanding of the molecular pathogenesis mechanisms, as this approach targets the underlying cause of the disorder – the impaired TCF4 and its lossof- function mutations. However, should gene therapy prove ineffective for humans in the postnatal period or be unfeasible in utero, the main focus of research would likely shift towards developing treatment strategies that target TCF4-regulated molecular pathways and downstream target genes.

## Conclusion

To date, substantial experimental data have accumulated, demonstrating the important role of the TCF4 transcription factor in the development and functioning of the nervous system. TCF4 structure and function anomalies are shown to drive the development of Pitt–Hopkins syndrome, and variants in the gene are associated with a number of psychiatric disorders. However, the molecular mechanisms behind these conditions remain unexplored, and our knowledge of the TCF4 upregulation and its downstream target genes is limited. Moreover, there is insufficient information on the dynamic expression and function of TCF4 during ontogenesis. It is also unclear how the activity of the encoded transcription factor changes depending on dimerization partners.

Given the broad expression pattern of TFC4, as well as its involvement in the development of the nervous system, it can be assumed that pathogenic variants affecting this gene may also be associated with other pathological conditions. This idea is supported by transcriptomic dynamics during TCF4 loss of function, as well as by studies of DNA-protein interactions using ChIP-Seq technology, indicating that common pathogenetic pathways seem to be involved in the pathogenesis of PTHS and some psychiatric disorders (Xia et al., 2018; Phan et al., 2020).

Although many aspects of TCF4 function remain to be explored, this transcription factor is evidently one of the key proteins responsible for learning, memory, verbal contact, and communicative functions in the context of psychiatric disorders. Further study of TCF4 and the identification of molecular pathways and target genes it regulates is crucial for understanding the pathogenesis of TCF4-associated diseases. This research direction is also important for finding potential therapeutic strategies for PTHS and possibly other socially significant diseases such as schizophrenia and bipolar disorder.

## Conflict of interest

The authors declare no conflict of interest.
